# Phenanthrene degradation by a flavoprotein monooxygenase from *Phanerodontia chrysosporium*

**DOI:** 10.1128/aem.01574-24

**Published:** 2025-02-03

**Authors:** Mika Hayasaka, Link Hamajima, Yuki Yoshida, Reini Mori, Hiroyuki Kato, Hiromitsu Suzuki, Ryoga Tsurigami, Takaaki Kojima, Masashi Kato, Motoyuki Shimizu

**Affiliations:** 1 Faculty of Agriculture, Meijo University 12942 , Nagoya, Japan; Royal Botanic Gardens, Surrey, United Kingdom

**Keywords:** phenanthrene, flavoprotein monooxygenase, intradiol dioxygenase, *Phanerochaete chrysosporium*, white-rot fungus

## Abstract

Phenanthrene (PHEN), a polycyclic aromatic hydrocarbon (PAH), is degraded by white-rot fungi like *Phanerochaete chrysosporium* (the fungus has been renamed as *Phanerodontia chrysosporium*). PHEN is metabolized by *P. chrysosporium* and transformed into various monohydroxylated and dihydroxylated products. These intermediates are further degraded by cleavage of the aromatic ring. However, the enzymes involved in PHEN conversion in *P. chrysosporium* remain largely unidentified. We aimed to identify and characterize the *P. chrysosporium* enzymes involved in the degradation of PHEN and its intermediates. Recombinant *P. chrysosporium* flavoprotein monooxygenase 11 (FPMO11), a homolog of the salicylate 1-monooxygenase from the naphthalene-degrading bacterium *Pseudomonas putida* G7, was overexpressed in *Escherichia coli*. FPMO11 catalyzes the oxidative decarboxylation of 1-hydroxy-2-naphthoate (1H2N) and 2-hydroxy-1-naphthoate (2H1N) to 1,2-dihydroxynaphthalene (1,2DHN). To the best of our knowledge, this is the first study to identify and characterize enzymes with 1H2N and 2H1N monooxygenase activities in members of the FPMO superfamily. Additionally, our search for a dioxygenase with the ability to catalyze the aromatic ring cleavage of 1,2DHN led to the identification of intradiol dioxygenase (IDD) 1 and IDD2 from *P. chrysosporium*, which catalyzes the ring cleavage of 1,2DHN. Thus, this study also identified, for the first time, intradiol 1,2DHN dioxygenase activity in members of the IDD superfamily. The findings highlight the unique substrate spectra of FPMO11 and IDDs, rendering them attractive candidates for biotechnological applications, especially mitigation of environmental and health risks associated with PAH pollution.

**IMPORTANCE:**

Phenanthrene (PHEN), a polycyclic aromatic hydrocarbon (PAH), is a widely studied pollutant in environmental science and toxicology due to its presence in fossil fuels, tobacco smoke, and as a byproduct of incomplete combustion processes. White-rot fungi like *P. chrysosporium* can degrade PHEN through the production of extracellular oxidative enzymes. We investigated the properties of PHEN-degrading enzymes in *P. chrysosporium*, specifically one flavoprotein monooxygenase (FPMO11) and two intradiol dioxygenases (IDD1 and IDD2). Our findings indicate that the enzymes catalyze the aromatic ring cleavage of PHEN, using the intermediates as substrates, transforming them into less harmful and more biodegradable compounds. This could help reduce environmental pollution and mitigate health risks associated with PAH exposure. The potential of these enzymes for biotechnological applications is also highlighted, emphasizing their critical role in understanding PAH degradation by white-rot fungi.

## INTRODUCTION

Fossil fuels for energy and raw material significantly contribute to environmental pollution and health issues by releasing pollutants during extraction, processing, and combustion, including polycyclic aromatic hydrocarbons (PAHs), which pose potential health risks due to their toxicity, persistence, and bioaccumulative nature (1). PAHs are a class of chemicals that occur naturally in coal, crude oil, and gasoline and are also produced by the incomplete combustion of fossil fuels, wood, garbage, and tobacco (2). The United States Environmental Protection Agency has classified 16 highly toxic, mutagenic, carcinogenic, teratogenic, and immunotoxic PAHs as priority pollutants due to their high concentrations, exposure, and toxicity (1, 2). Among the 16 PAHs, phenanthrene (PHEN) is a low-molecular-weight water-soluble compound that significantly affects organisms through various mechanisms including aryl hydrocarbon receptor activation and reproductive endocrine disruption (3). PHEN, containing three fused benzene rings, is generated by the combustion of fossil fuels, various industrial processes, and natural events such as forest fires (2). PHEN has been used as a model compound to investigate the biodegradation of PAHs because (i) it is found in high concentrations in PAH-contaminated environmental samples and (ii) its potential carcinogenicity.

White-rot fungi are capable of degrading a wide variety of recalcitrant aromatic compounds, including lignin, as well as environmentally persistent pollutants such as PAHs, primarily due to their production of extracellular oxidative enzymes, such as lignin peroxidases (LiPs), manganese peroxidases (MnPs), and laccases (4–11). These nonspecific one-electron oxidizing enzymes cleave the carbon-carbon bonds and the oxygen-carbon bonds in aromatic compounds, resulting in the conversion and reduced toxicity of various aromatic compounds such as PAHs (11). *Phanerochaete chrysosporium* (renamed as *Phanerodontia chrysosporium*) is one of the best-studied white-rot basidiomycetes and also produces LiP and MnP (7–10); bioconversion of PHEN by *P. chrysosporium* has been extensively studied (12–14). The fungus can degrade PHEN, oxidizing it initially to phenanthrene-9,10-quinone, and then to the ring-cleavage product 2,2’-diphenic acid (DPA) under ligninolytic conditions (6). Furthermore, it has been shown that LiP and MnP can oxidize 9-phenanthrol (9PL) to DPA via lipid peroxidation (13, 15). Notably, under non-ligninolytic conditions (no production of LiP or MnP), *P. chrysosporium* metabolizes PHEN to phenanthrene *trans*-3,4- and *trans*-9,10-dihydrodiols (3,4PDL and 9,10PDL, respectively); 3-, 4-, and 9-phenanthrols (3PL, 4PL, and 9PL, respectively); and a glucoside conjugate of 9PL (14). Thus, *P. chrysosporium* contains several enzymatic pathways implicated in PHEN metabolism (13). However, the enzymes involved in PHEN conversion in *P. chrysosporium* remain largely unidentified.

Flavoprotein monooxygenases (FPMOs) are a diverse group of enzymes widely distributed in living organisms. They are involved in various biological processes such as drug detoxification, biodegradation of environmental aromatic compounds, and biosynthesis of antibiotics (16). Hydroxylation reactions mediated by FPMOs play an important role in aromatic compound metabolism in prokaryotes and eukaryotes (16). Based on their structural and functional properties, FPMOs are divided into eight groups (A–H) (16). Several bacterial species capable of degrading PHEN have been identified (17–22). PHEN is converted to 1-hydroxy-2-naphthoate (1H2N), which is subsequently oxidatively decarboxylated to 1,2-dihydroxynaphthalene (1,2DHN) by monooxygenases, including FPMOs (17–22). The genome sequence of *P. chrysosporium* revealed the genetic diversity of FPMOs, identifying as many as 57 FPMO-related genes (https://mycocosm.jgi.doe.gov/Phchr4_2/Phchr4_2.home.html), suggesting that some FPMOs might possess the PHEN and its metabolite conversion activity.

In this study, we aimed to investigate the degradation of PHEN and its intermediates by *P. chrysosporium*. Furthermore, a FPMO and dioxygenases from *P. chrysosporium* were produced as recombinant proteins in *Escherichia coli* and characterized. The findings were analyzed with respect to enzymatic degradation of PHEN by *P. chrysosporium*.

## RESULTS

### Fungal metabolism of PHEN

**Fig 1:**
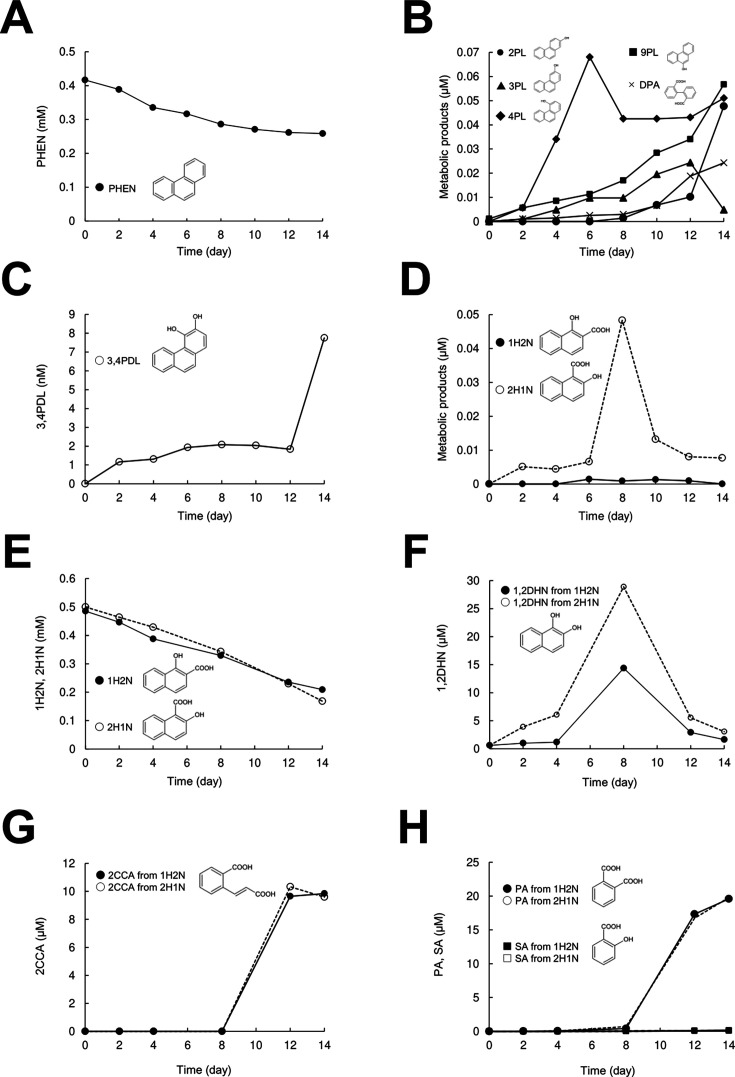
Fungal metabolism of PHEN. (**A**) Time course of PHEN conversion. After a 2-day pre-incubation, PHEN was added to a final concentration of 0.5 mM; PHEN ●. (**B, C, and D**) The metabolites of PHEN in the culture were identified using gas chromatography-mass spectrometry (GC-MS) with authentic standards as references. After 14 days of incubation with PHEN, metabolites of PHEN were identified using GC-MS. (**B**) 2-Phenanthrol (2PL, ●); 3-phenanthrol (3PL, ▲); 4-phenanthrol (4PL, ♦); 9-phenanthrol (9PL, ■); DPA (**×**). (**C**) 3,4-Dihydrodiols (3,4PDL, ○). (**D**) 1-Hydroxy-2-naphthoate (1H2N, □); 2-hydroxy-1-naphthoate (2H1N, △). (**E**) The time course of 1H2N or 2H1N conversion. After a 2-day pre-incubation, 1H2N or 2H1N was added to a final concentration of 0.5 mM. (1H2N, □; 2H1N, △). (**F, G, and H**) The metabolites of 1H2N or 2H1N in the culture were identified using GC-MS with reference to authentic standards. After 14 days of incubation with 1H2N or 2H1N, metabolites of 1H2N or 2H1N were identified using GC-MS. (**F**) 1,2-Dihydroxynaphthalene (1,2-DHN from 1H2N, ●; 1,2-DHN from 2H1N, ○). (**G**) 2-Carboxycinnamic acid (2CCA from 1H2N, ●; 2CCA from 2H1N, ○). (**H**) Phthalic acid (PA from 1H2N, ●; PA from 2H1N, ○); salicylic acid (SA from 1H2N, ■; SA from 2H1N, □). Data are presented as mean values of three independent experiments. The SEs were <19%.

Evaluation of the metabolism of PHEN by *P. chrysosporium* revealed a decline in its concentration from an initial value of 0.5–0.27 mM after 14 days of culture (Fig. 1A). Mono-hydroxylated products (4PL, 9PL, 3PL, and 2PL) and di-hydroxylated products (3,4PDL, DPA, 1H2N, and 2-hydroxy-1-naphthoate [2H1N]) were identified as fungal metabolic products of PHEN (Fig. 1B through D; Fig. S1). The concentrations of 1H2N and 2H1N decreased from an initial 0.5–0.2 mM in the medium following 14 days of cultivation indicating that these substrates were metabolized by *P. chrysosporium* (Fig. 1E). Additionally, 1,2DHN, 2-carboxycinnamic acid (2CCA), phthalic acid (PA), and salicylic acid (SA) were identified as fungal metabolic products of 1H2N and 2H1N (Fig. 1F through H; Fig. S1). These results showed that PHEN was hydroxylated to 3,4PDL via the intermediate synthesis of mono-hydroxylated PHEN and then transformed to 1H2N and 2H1N. The intermediates 1H2N and 2H1N are subsequently decarboxylated to 1,2DHN, which undergoes ring cleavage to form 2CCA. This product was further converted to PA and SA (Fig. S2).

Next, to elucidate the metabolic pathway and identify potential rate-limiting steps in the PHEN degradation by *P. chrysosporium*, the metabolism of intermediates was monitored. Due to the unavailability or prohibitively high cost of mono-hydroxylated PHEN derivatives (4PL, 9PL, 3PL, and 2PL) and di-hydroxylated PHEN derivatives (3,4PDL, 3,4PDL, and 1,2PDL), their direct metabolism by the fungus could not be assessed. However, intermediates, such as 1H2N, 2H1N, and 2CCA, were metabolized more rapidly to PHEN (Fig. 1; Fig. S1). Although PA was minimally degraded by the fungus, SA was identified as a metabolite (Fig. S2), suggesting that PA was, at least partially, converted to SA by the fungus (Fig. 1; Fig. S2). Notably, the addition of 0.5 mM SA—known to exhibit anti-fungal properties—to the culture inhibited fungal growth. Nonetheless, the conversion ratio of SA by the fungus was comparable to that of PHEN. Moreover, catechol (CAT) was identified as a metabolite of SA (Fig. S2). These observations indicate that the monooxygenation and/or dioxygenation reactions of PHEN and the conversion of PA may represent the rate-limiting steps in the PHEN degradation by *P. chrysosporium*. The proposed metabolic pathway is summarized in Fig. S3.

In the present study, we further aimed to identify the proteins in *P. chrysosporium* involved in the decarboxylation of 1H2N and 2H1N.

### Search for a hydroxynaphthoate monooxygenase from *P. chrysosporium* genome

As hydroxynaphthoates such as 1H2N and 2H1N are derivatives of SA, SA 1-monooxygenase belonging to FPMO group A from *P. chrysosporium* may catalyze the decarboxylation reaction of the derivatives. To identify hydroxynaphthoate monooxygenase candidates, the publicly available *P. chrysosporium* genome (database version 4; https://mycocosm.jgi.doe.gov/Phchr4_2/Phchr4_2.home.html) was screened via BLAST using the amino acid sequence of salicylate 1-monooxygenase (NahG) from the PAH-degrading bacterium *Pseudomonas putida* G7 (Protein ID P23262) as a reference (23–25). Although it remains unclear whether NahG catalyzes the decarboxylation of 1H2N and/or 2H1N, its selection as a reference in the present study was based on the known ability of *Pseudomonas* species, including *P. putida* G7, to degrade PHEN (23–25). NahG was selected as a reference in the present study. The BLAST search results revealed that NahG shares 49.4% amino acid sequence identity with FPMO11 (Protein ID 6385018) from *P. chrysosporium*. Figure S4 shows the sequence alignment of FPMO11 with well-characterized SA 1-monooxygenases, including NahG, ShyA (A2QWH1) from *Aspergillus niger* (26), and SalA (Q9HFQ8) from *Aspergillus nidulans* (27). The histidine residue (H238) involved in deprotonation of SA, glycine (G43, G45, G47, G178, and G329), aspartate (D176 and D330), arginine (R125), and glutamate (E34) residues involved in flavin adenine dinucleotide (FAD) retention, phenylalanine residues (F242 and F255) that increase the hydrophobicity of the substrate pocket, and the charged residues (H338 and D397) that interact with the substrate were highly conserved (Fig. S4).

### Catalytic conversion of salicylate derivatives by FPMO11

**Fig 2:**
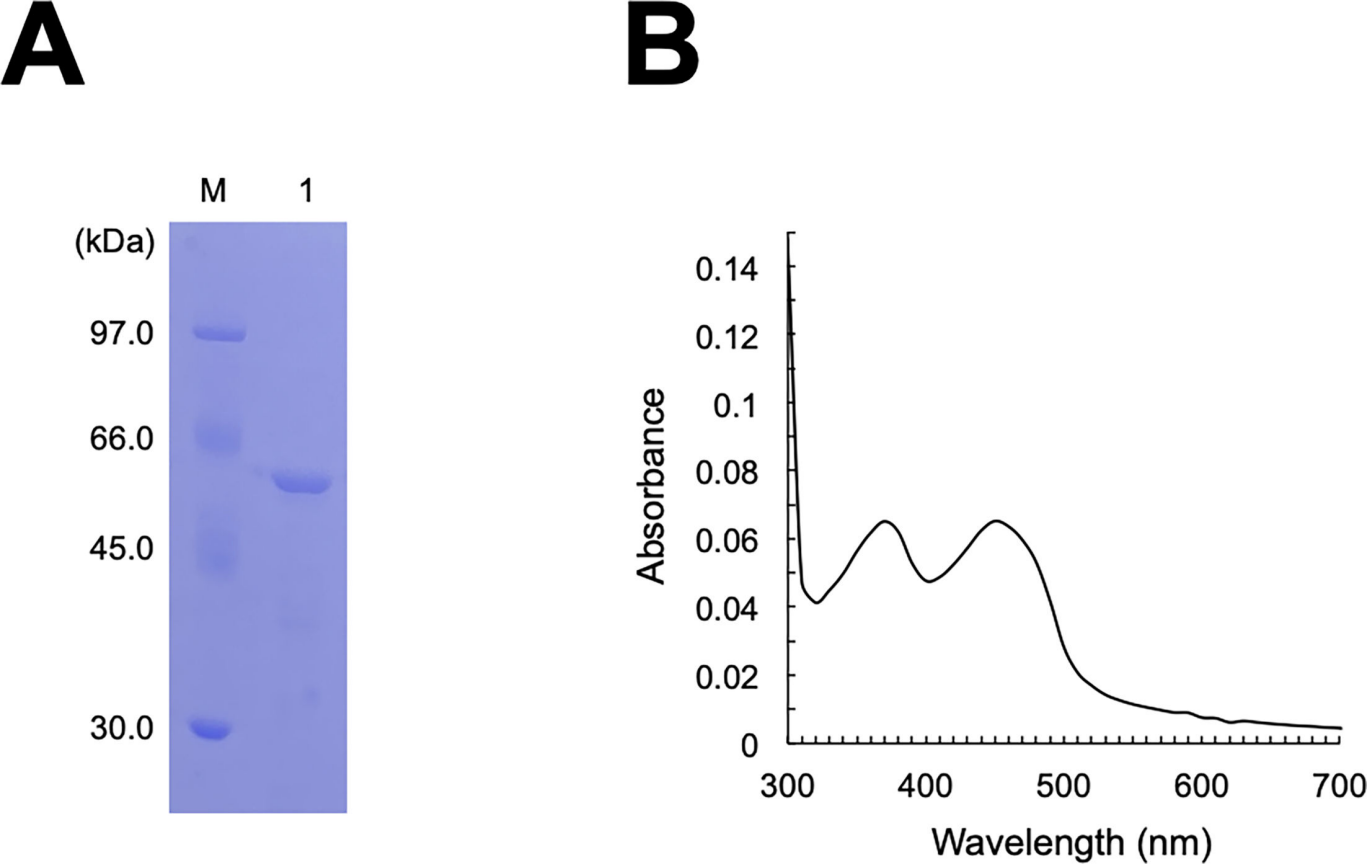
SDS-PAGE analysis and absorption spectrum of recombinant FPMO11. (**A**) SDS-PAGE analysis of purified *P. chrysosporium* flavoprotein monooxygenase 11 (FPMO11). lane 1, FPMO11; lane M, protein molecular mass markers. (**B**) Absorption spectra of FPMO11.

The C-terminal 6 × His-tagged FPMO11 was produced in *E. coli*, and the molecular weight (47.5 kDa) of the protein band was consistent with that predicted from the deduced amino acid sequence (Fig. 2A). The UV-visible scan of the purified recombinant FPMO11 showed a typical spectrum profile derived from flavin prosthetic groups, with absorption peaks at 380 nm and 450 nm (Fig. 2B).

**Fig 3:**
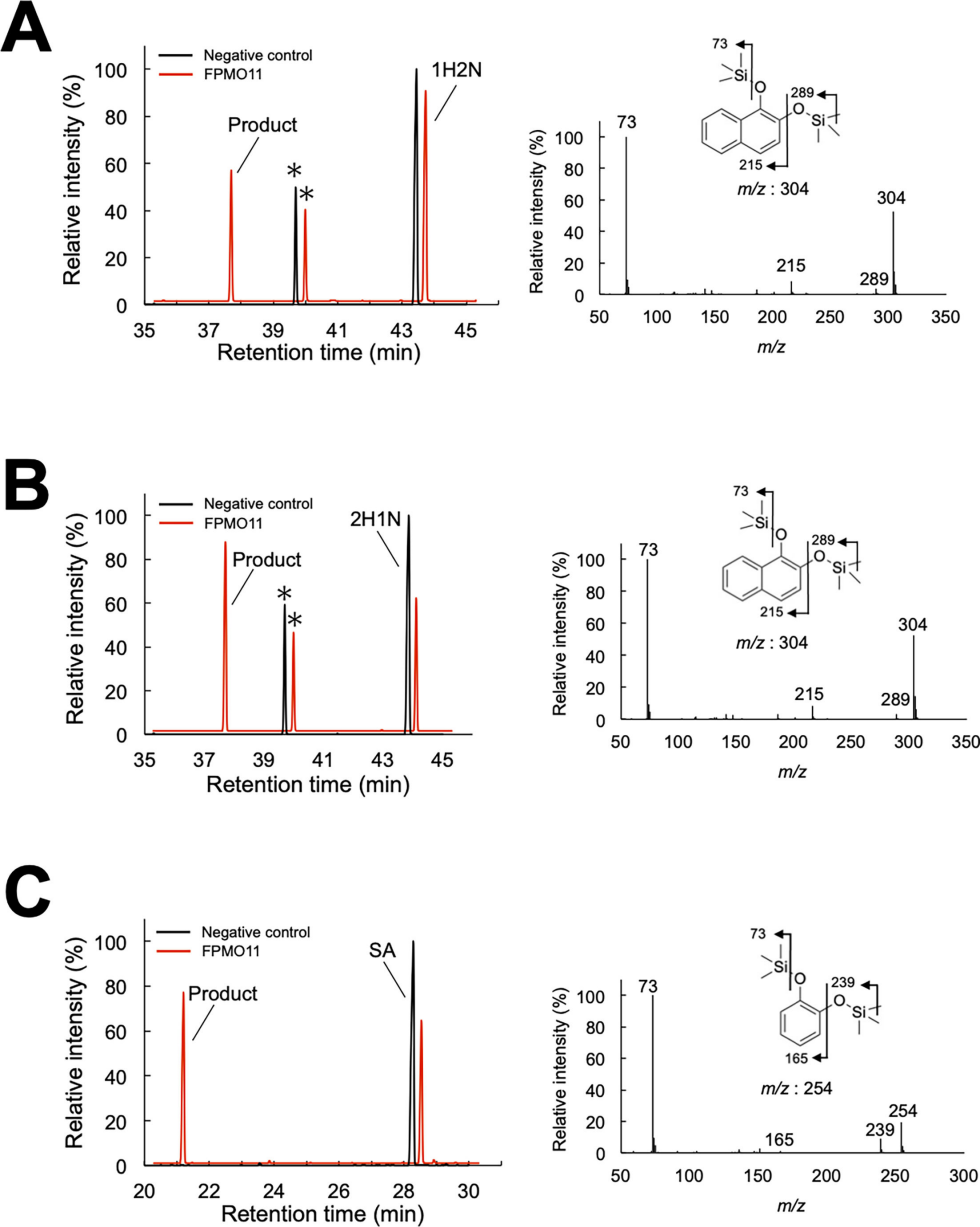
Total ion chromatograms and mass spectra of the reaction products generated by *P. chrysosporium* flavoprotein monooxygenase 11 (FPMO11) from 1H2N (**A**), 2H1N (**B**), and SA (**C**) as substrates. The TMS-derivatized reaction products were analyzed by GC-MS. Mass spectra (A, B, 1,2-dihydroxynaphtharene [1,2DHN]; C, catechol [CAT]) of the reaction products were obtained from the GC peaks appearing at retention times of 37.6 min (**A and B**) and 21.2 min (**C**). The asterisks indicate contaminants. The experiment was performed three times, and representative results are shown.

**Fig 4:**
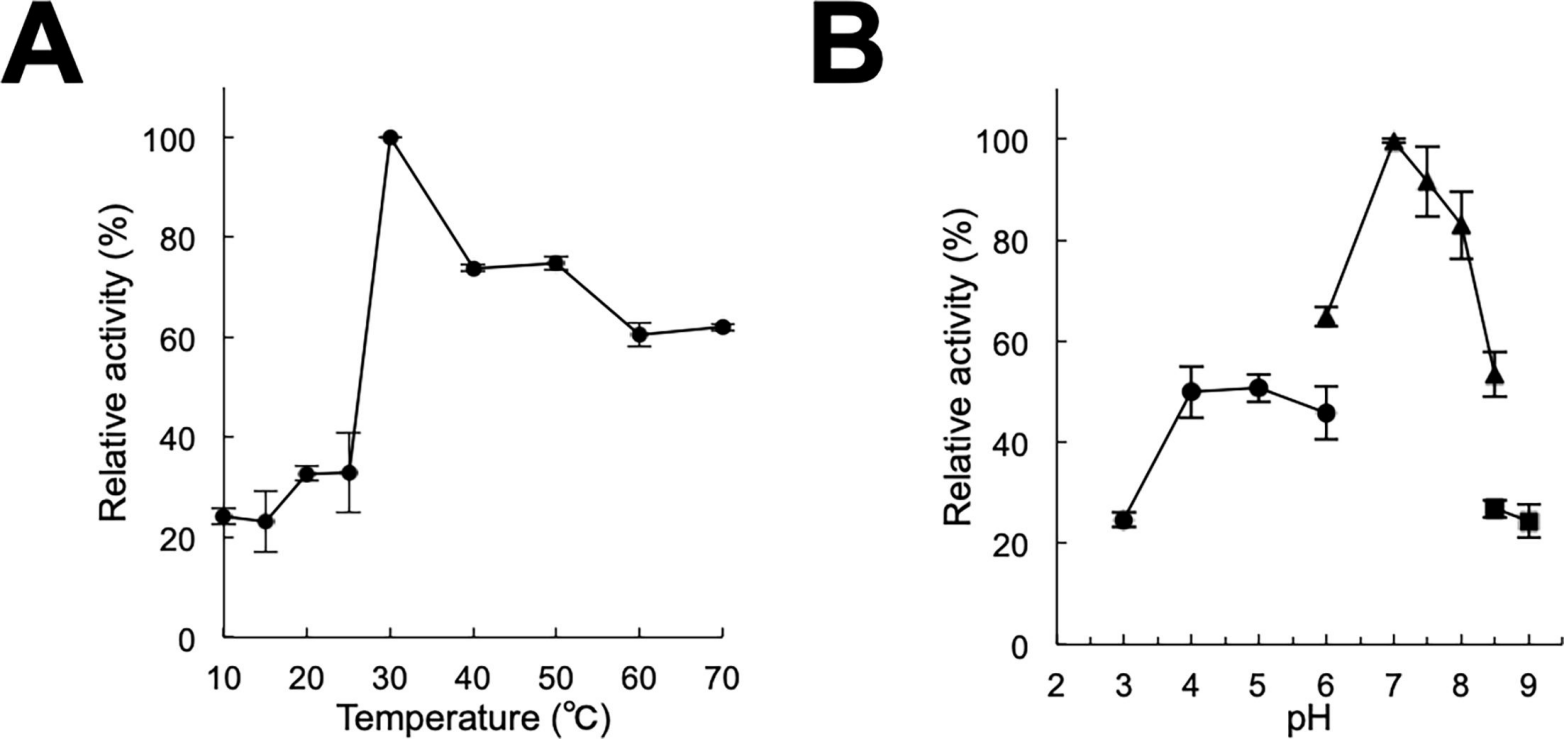
Optimal temperature and pH of FPMO11. (**A**) Optimal temperature of *P. chrysosporium* flavoprotein monooxygenase 11 (FPMO11) using SA as a substrate. Enzyme reactions proceeded at temperatures ranging from 20°C to 70°C. (**B**) Optimal pH of FPMO11. Enzyme reactions proceeded over the pH range 3.0–9.0: in 50 mM sodium acetate (pH 3.0–6.0; ●), 50 mM HEPES-NaOH (pH 6.0–8.5; ▲), and 50 mM Tris-HCl (pH 8.5–9.0; ■). Data are presented as mean ± SD of four experiments.

The catalytic conversions of 2H1N, 1H2N, and SA were analyzed, using NADPH as a co-substrate, to evaluate the enzymatic activity of recombinant FPMO11. The decarboxylated products of 2H1N, 1H2N, and SA were determined using gas chromatography-mass spectrometry (GC-MS) analysis (Fig. 3). The mass spectra of the trimethylsilyl (TMS) derivatives of the reaction product showed the same fragmentation patterns as that of 1,2DHN and CAT (Fig. 3). Subsequently, several SA derivatives were tested as substrates for FPMO11. The decarboxylated products of the SA derivatives gentisate (GA), 2,3-dihydroxybenzoate (2,3DHB), 2,4-dihydroxybenzoate (2,4DHB), 4-chlorosalicylate (4CSA), 2-hydroxy-3-phenylbenzoate (2H3PB), and 2-hydroxy-6-phenylbenzoate (2H6PB) converted by FPMO11 were identified using GC-MS (Fig. S5). FPMO11 decarboxylated these substrates to 1,2,4-trihydroxybenzene (1,2,4THB), pyrogallol (PG), 1,2,4THB, 4-chlorocatechol (4CCAT), biphenyl-2,3-diol (B2,3D), and B2,3D (Fig. S5). Overall, FPMO11 catalyzed the formation of nine SA derivatives, including 1H2N and 2H1N (Fig. S6). The optimal reaction condition for the activity of FPMO11 was determined using SA as the substrate and found to be 30°C and pH 7.0 (Fig. 4A and B).

To compare the substrate-specificity with other SA 1-monooxygenase, a recombinant C-terminal 6 × His-tagged NahG (P23262) from *P. putida* was overexpressed in *E. coli* (Fig. S7). Catalytic conversion of the nine SA derivatives was analyzed by GC-MS to evaluate the substrate specificity of recombinant NahG using NADH as a co-substrate. GC-MS analysis showed the products from the decarboxylation of SA, GA, 2,3DHB, 2,4DHB, and 4CSA by NahG (Fig. S8); whereas no conversion products of 2H1N, 1H2N, 2H3PB, or 2H6PB were observed. These results indicate that the activity of NahG toward the four SA derivatives was significantly lower than that of FPMO11 (Fig. S8). These findings also reveal the uniquely broad substrate spectrum of FPMO11 compared with that of NahG.

### Apparent kinetic parameters of FPMO11

The apparent kinetic parameters of FPMO11 and NahG were investigated using the SA derivatives and NAD(P)H as substrates and co-substrates, respectively (Table 1). FPMO11 was active against nine SA derivatives, and the highest catalytic efficiency (*k_cat_*/*K_m_*) was observed for SA. The lowest *K_m_* value was observed for SA among all substrates, whereas the highest *k_cat_* was obtained with 2,4DHB. The *k_cat_*/*K_m_* value for 2H1N was 3.4-fold higher than that for 1H2N. NahG exhibited activity against five SA derivatives, and the highest *k_cat_*/*K_m_* was observed for SA. The lowest *K_m_* was also observed for SA, whereas the highest *k_cat_* was obtained for GA. The *k_cat_*/*K_m_* value of NahG for monoaromatics, such as SA, GA, and 2,4DHB, was slightly higher than that of FPMO11.

**TABLE 1:** Apparent kinetic parameters of FPMO11 and NahG toward SA derivatives^*a*^^*c*^

	FPMO11			NahG		
Substrate	*K_m_* (μM)	*k_cat_* (min^−1^)	*k_cat_/K_m_* (min^−1^/μM)	*K_m_* (μM)	*k_cat_* (min^−1^)	*k_cat_/K_m_* (min^−1^/μM)
SA	30.0 ± 34	190 ± 22	6.4	28 ± 3.0^*b*^	200 ± 6.0^*b*^	7.1^*b*^
1H2N	100 ± 15	16 ± 3.7	0.16	ND	ND	ND
2H1N	110 ± 25	58 ± 0.64	0.54	ND	ND	ND
GA	48 ± 45	150 ± 9.6	3.1	110 ± 30	420 ± 42	3.7
2,3DHB	67 ± 1.8	190 ± 1.9	2.9	120 ± 15	330 ± 34	2.7
2,4DHB	82 ± 9.7	320 ± 37	3.8	70 ± 15	310 ± 28	4.4
4CSA	120 ± 28	170 ± 18	1.4	51 ± 19	55 ± 24	1.1
2H3PB	290 ± 67	15 ± 1.8	0.051	ND	ND	ND
2H6PB	320 ± 84	6.4 ± 1.8	0.020	ND	ND	ND
NADPH	150 ± 37	320 ± 30	2.2	69 ± 17	80 ± 35	1.2
NADH	170 ± 60	220 ± 24	1.3	27 ± 10	120 ± 37	4.3

^*a*^ The activity levels of FPMO11 were determined in the reaction mixtures (0.1 mL) containing 0.1–5 μM FPMO11 and 5μL of substrate solutions (0–600 mM in dimethylsulfoxide) in 50 mM HEPES-NaOH (pH 7.0) at 30°C. We used the initial velocity 30 s after the addition of each substrate to calculate the apparent kinetic parameters. Data are presented as mean ± SE of three experiments. ND indicates no activity.

^*b*^ Costa et al. (23).

^*c*^ Abbreviations: FPMO11, flavoprotein monooxygenase 11 from *P*. *chrysosporium*; NahG; salicylate 1-monooxygenase; 1H2N, 1-hydroxy-2-naphthoate; 2H1N, 2-hydroxy-1-naphthoate; GA, gentisate; 2,3DHB, 2,3-dihydroxybenzoate; 2,4DHB, 2,4-dihydroxybenzoate; 4CSA, 4-chlorosalicylate; 2H3PB, 2-hydroxy-3-phenylbenzoate; 2H6PB, 2-hydroxy-6-phenylbenzoate.

### Predicted structure of FPMO11

**Fig 5:**
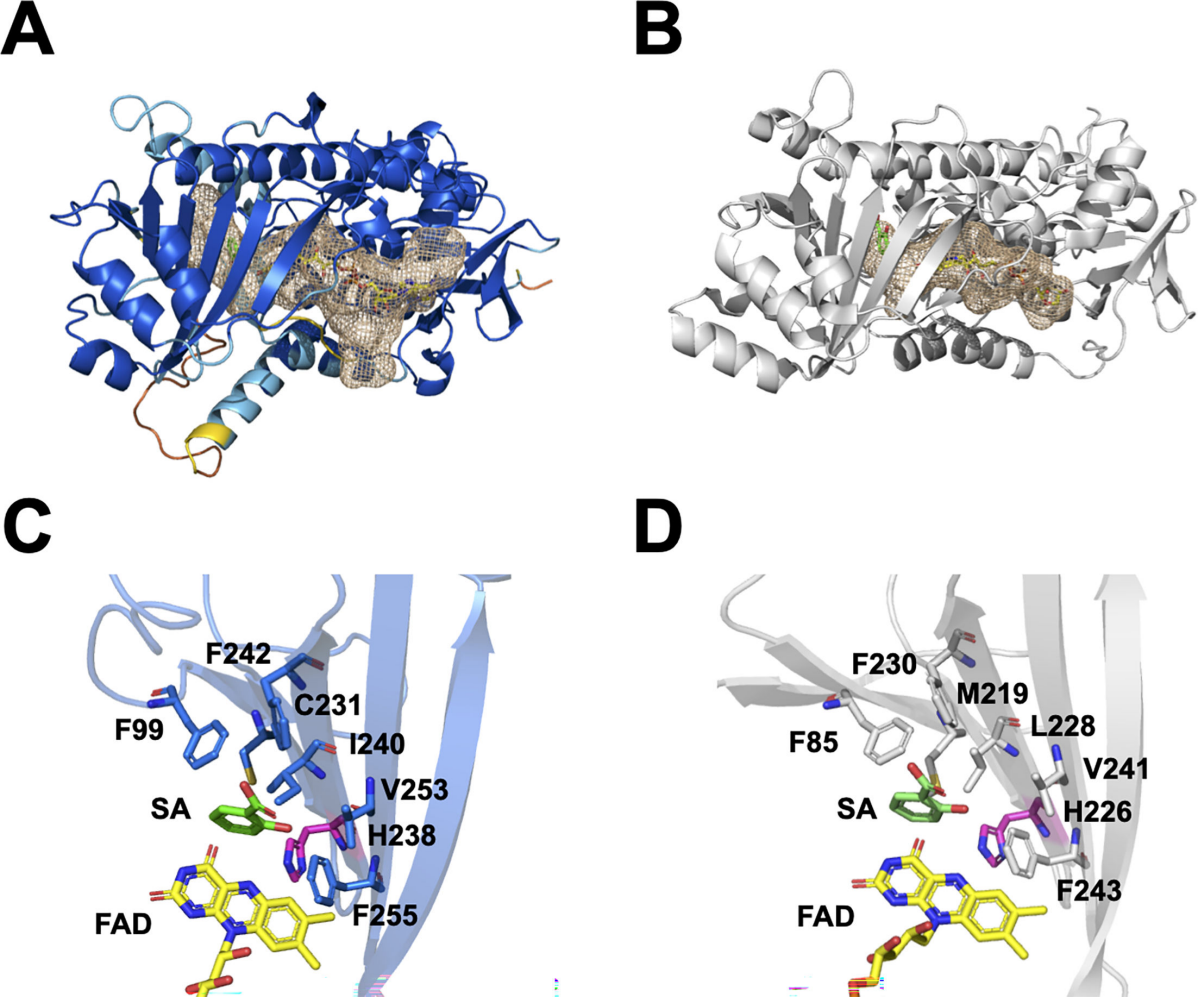
Structural comparison of FPMO11 and NahG. (**A and B**) The structures of flavoprotein monooxygenase 11 from *P. chrysosporium* (FPMO11) (**A**) predicted using AlphaFold2 (navy blue indicates the pLDDT score <100; cyan indicates the pLDDT score <90; yellow indicates the pLDDT score <70; orange indicates the pLDDT score <50; yellow stick indicates flavin adenine dinucleotide [FAD]) and salicylate 1-monooxygenase (NahG) crystal structure (PDB ID: 5EVY) from *P. putida* G7 (**B**). (**C and D**) Active-sites of FPMO11 docked with salicylic acid (SA) and FAD (green and yellow sticks indicate SA and FAD, respectively) using AlphaFill (**C**) and NahG crystal structure liganded with SA (PDB ID: 5EVY) (**D**). Histidine residues (purple sticks) are responsible for substrate deprotonation for catalytic activity.

Structure prediction of FPMO11 was performed using the AlphaFold2 (28) and AlphaFill (29) programs. The obtained model was used for structural comparison with the NahG crystal structure (PDB ID: 5EVY) liganded with SA (23) (Fig. 5). Although the amino acid sequence identity between NahG and FPMO11 was 49.4% (Fig. S4), both proteins displayed a highly conserved overall fold, presence of FAD, and the combination of α-helices and β-sheets in the substrate-binding domain (Fig. 5A and B ). The root mean square deviation between the crystal structure of NahG and the predicted structure of FPMO11 was 1.248, indicating a high similarity between the two structures. The putative general base that promotes substrate activation in NahG is His226, which is conserved as His238 in FPMO11 (Fig. 5C and D). Despite this well-conserved overall fold, the local similarity data prediction showed that some parts of the enzyme models, mostly the flexible loops, had low confidence level (Fig. 5A and B ).

**Fig 6:**
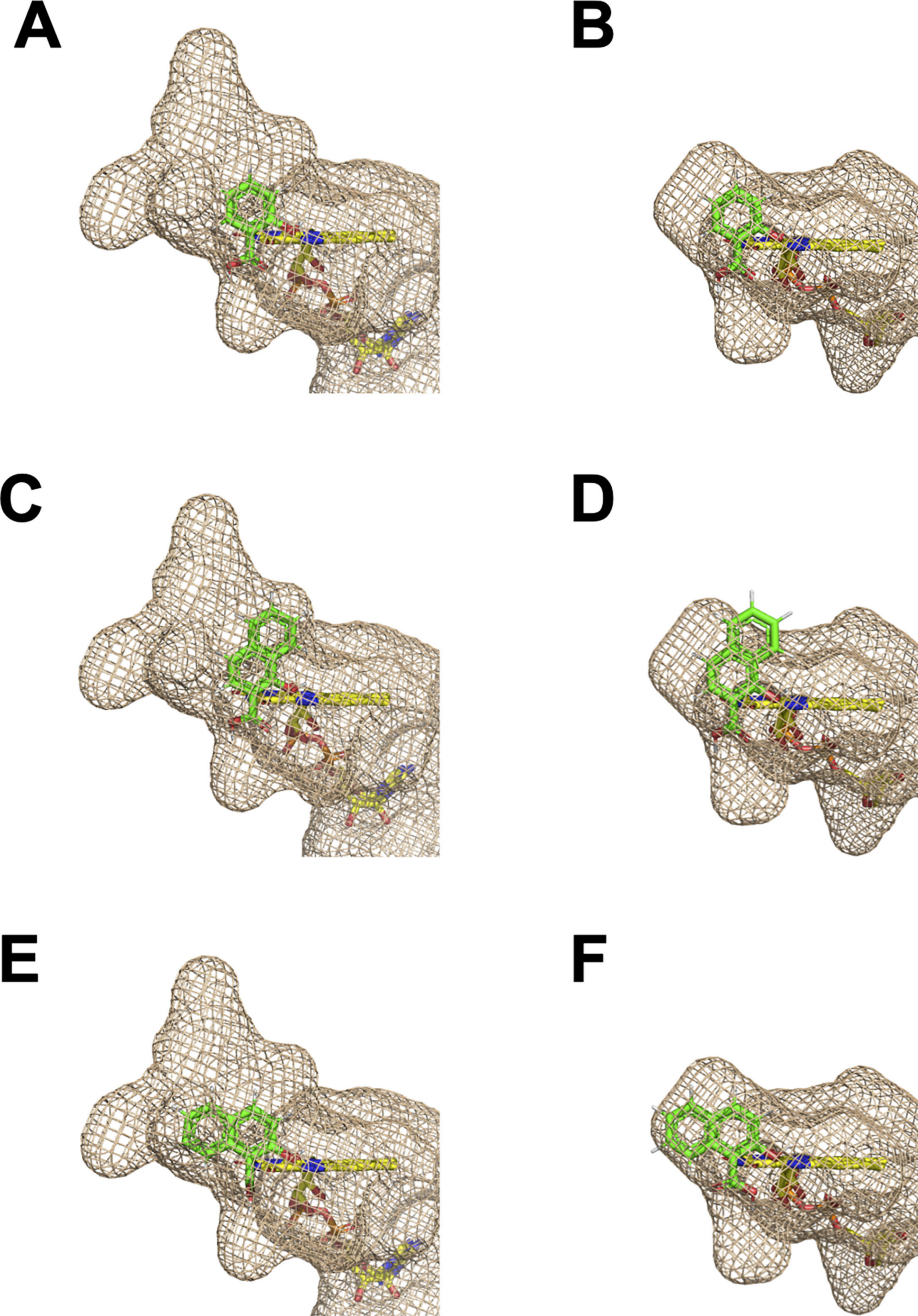
Comparison of the cavities in the active-site pockets of FPMO11 and NahG. The cavities in the active-site pockets of flavoprotein monooxygenase 11 from *P. chrysosporium* (FPMO11) (A, C, and E) and salicylate 1-monooxygenase (NahG) (B, D, and F) with the substrates are indicated by a brown mesh. SA docked in the active-site pockets of FPMO11 (**A**) and NahG (**B**). 1-Hydroxy-2-naphthoate (1H2N) docked in the active-site pocket of FPMO11 (**C**) and NahG (**D**). 2-Hydroxy-1-naphthoate (2H1N) docked in the active-site pocket of FPMO11 (**E**) and NahG (**F**). Green and yellow sticks indicate the substrates (1H2N and 2H1N) and FAD, respectively.

We utilized the AlphaFill program (29) to predict the binding sites in the FPMO11 structure. FAD (PDB ID: 6NET) and SA (PDB ID: 5EVY) were predicted to be ligands for FPMO11 (Fig. 5C). The SA-binding models showed similar orientations for both the enzymes, suggesting that the prediction of the ligand at the binding site was almost accurate (Fig. 6A and B ). When 1H2N and 2H1N were positioned at the same location as SA, the substrates docked well into the active-site pocket of FPMO11 (Fig. 6C and E). 1H2N and 2H1N could not be docked into the pocket of NahG (Fig. 6D and F) since the cavity in the active site pocket of NahG was smaller than that of FPMO11. These results indicate that the size of the cavity in the active site pocket of FPMO11 affects substrate specificity toward aromatic dimers, such as 1H2N and 2H1N.

### Ring cleavage of 1,2DHN by IDD1 and IDD2, members of intradiol dioxygenase superfamily

**Fig 7:**
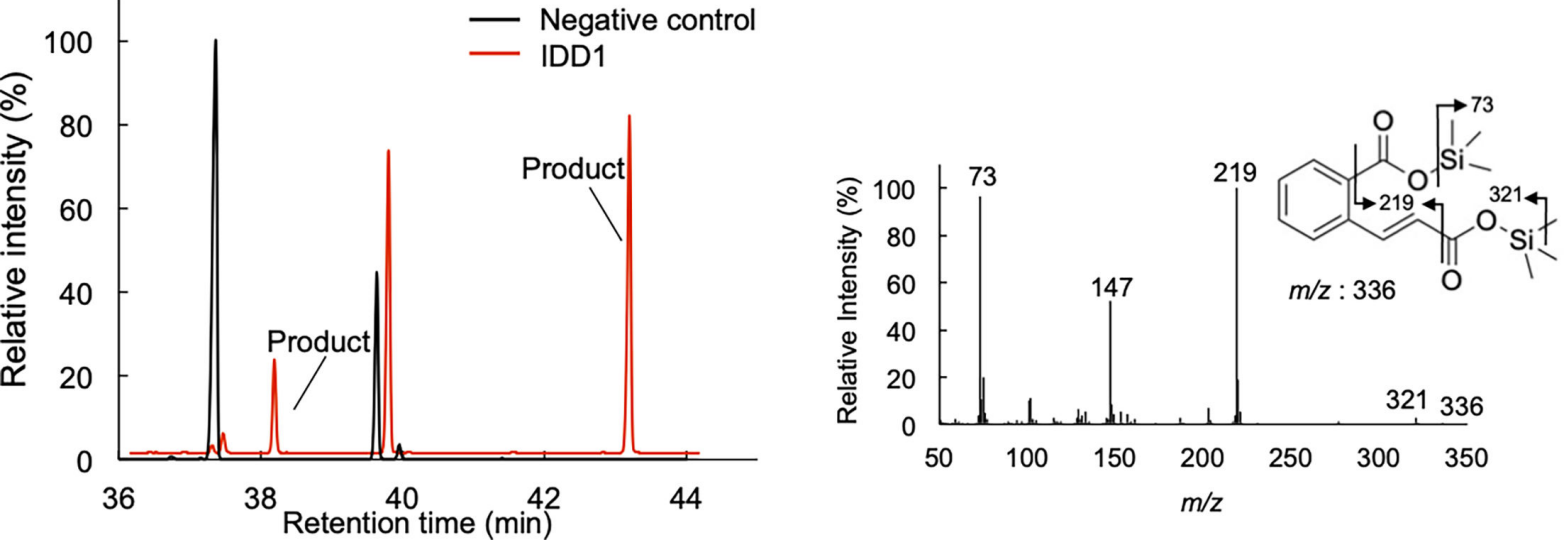
Total ion chromatogram and mass spectrum of the reaction product generated by *P. chrysosporium* intradiol dioxygenase (IDD1) from 1,2DHN. The TMS-derivatized reaction products from 1,2-dihydroxynaphthalene (1,2DHN) were analyzed using GC-MS. The mass spectrum of reaction products was obtained from the GC peaks appearing at retention times 38.2 and 43.3 min. The experiment was performed three times, and representative results are shown.

We recently identified methoxytrihydroxybenzene dioxygenases, IDD1 and IDD2, belonging to the intradiol dioxygenase (IDD) superfamily, in *P. chrysosporium* (30). In particular, IDD1 has a broad substrate specificity toward CAT derivatives (30). Thus, we evaluated whether 1,2DHN could be a substrate for both IDD1 and IDD2 (Fig. S9). The reaction products obtained from the ring cleavage of 1,2DHN by IDD1 were determined by GC-MS analysis (Fig. 7). The mass spectra of the TMS derivatives of the reaction products show fragmentation patterns identical to those of 2CCA (Fig. 7). Similar results were obtained for IDD2 (data not shown). The apparent kinetic parameters of IDD1 and IDD2 were investigated using 1,2DHN as the substrate (Table S1). The rates of O_2_ consumption by IDD1 and IDD2 in the presence of 1,2DHN were measured by monitoring the changes in O_2_ concentration polarographically. The *k_cat_*/*K_m_* value of IDD1 with 1,2DHN was higher than that of IDD2 (Table S1). This is the first study to identify and characterize 1,2DHN dioxygenase activities in members of the IDD superfamily.

## DISCUSSION

**Fig 8:**
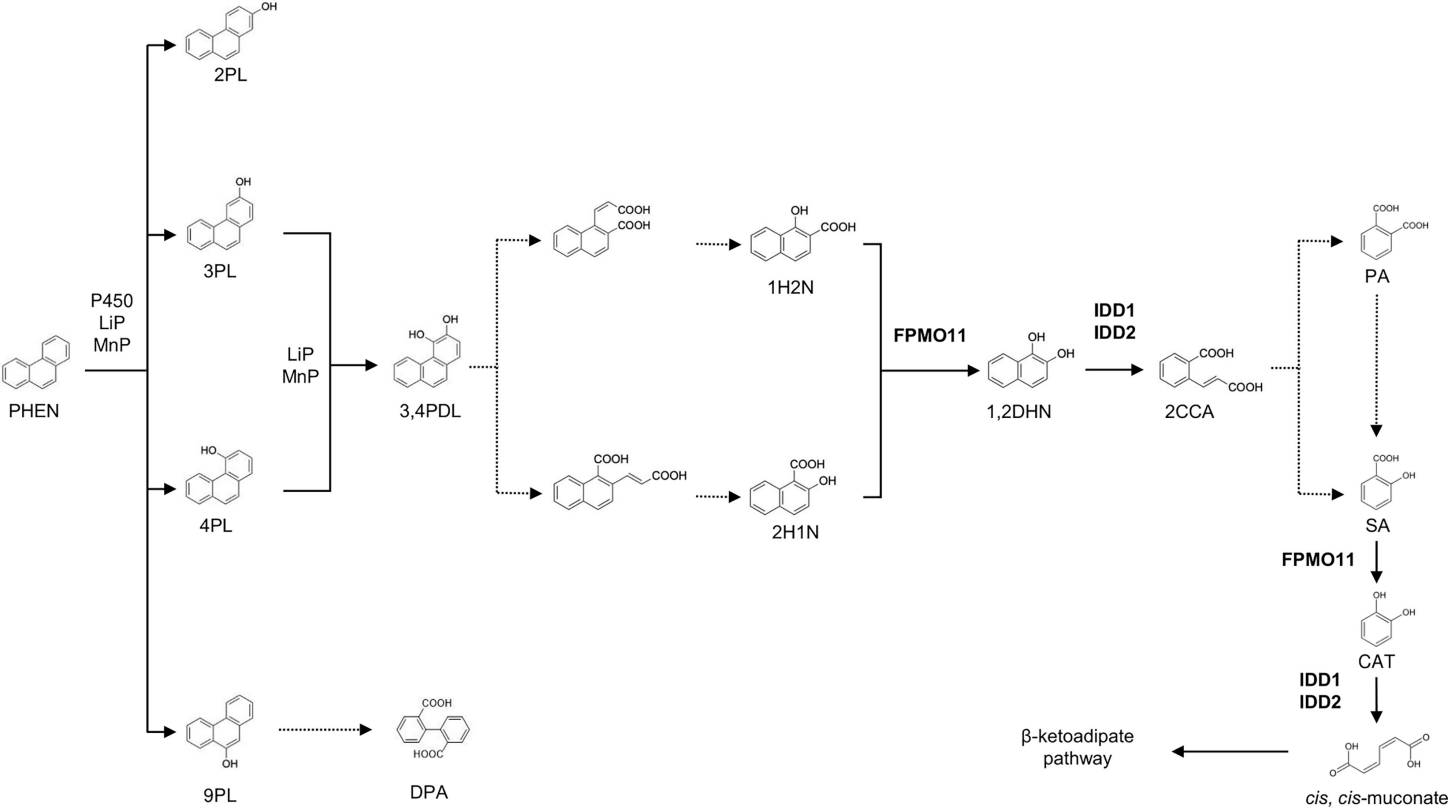
Metabolic pathway of PHEN in the white-rot fungus *P. chrysosporium*. Dotted arrows indicate the estimated reactions; solid arrows indicate the reactions of the identified enzymes: cytochrome P450 monooxygenases (Pc-Pah4 and Pc-Pah6), LiP, MnP, flavoprotein monooxygenase 11 from *P. chrysosporium* (FPMO11), and intradiol dioxygenases from *P. chrysosporium* (IDD1 and IDD2).

In this study, we report the conversion of PHEN and its intermediates by the white-rot fungus *P. chrysosporium*. FPMO11 was capable of the oxidative decarboxylation of nine SA derivatives, including PHEN intermediates such as 1H2N and 2H1N (Fig. S6). Additionally, IDD1 and IDD2 catalyzed the ring cleavage of 1,2DHN (Fig. 7). These results indicate that the enzymes play important roles in PHEN degradation by *P. chrysosporium* (Fig. 8).

LiP and MnP produced by *P. chrysosporium* under ligninolytic conditions convert PHEN to PHEN-9,10-quinone and then to the ring-cleavage product DPA (12, 13, 15). In addition, the fungus metabolizes PHEN to 3,4PDL; 9,10PDL; 3PL; 4PL; and 9PL under non-ligninolytic conditions (14). In the current study, we observed that *P. chrysosporium* converts PHEN to 1H2N and 2H1N (Fig. 1). These findings indicate that *P. chrysosporium* has multiple pathways for PHEN conversion.

Several bacterial species capable of degrading PHEN have been reported (17–22), in which the metabolic pathway is initiated by a ring-hydroxylating dioxygenase and/or a coupling reaction between monooxygenase and epoxide hydrolase to generate 3,4PDL, which is subsequently converted to 1H2N (17–22). 1H2N is metabolized through one of two distinct routes: the “phthalic acid” or the “naphthalene” (17, 18). In the “naphthalene” route of PHEN degradation, 1H2N is oxidatively decarboxylated to 1,2DHN by FPMOs. The 1,2DHN produced is then subject to ring cleavage by further enzymatic action, leading to the breakdown of the aromatic ring structure (17). Although the enzymes involved in PHEN conversion remain largely undetermined, nonligninolytic fungi, such as *Trichoderma* spp., have the ability to degrade PAHs to carbon dioxide (31). In addition to these microorganisms, *P. chrysosporium* metabolizes PHEN to 3PL, 4PL, and 9PL via the cytochrome P450 monooxygenases Pc-Pah4 and Pc-Pah6 (32). Although some bacteria such as *Pseudomonas* sp. convert PHEN to 1H2N (17, 18), *P. chrysosporium* converts PHEN to 1H2N as well as 2H1N. Additionally, FPMO11 catalyzed the oxidative decarboxylation of 1H2N and 2H1N to 1,2DHN, which is subsequently converted to 2CCA, PA, and SA (Fig. 8). These results show that PHEN is hydroxylated to 3,4PDL via mono-hydroxylated intermediates before being transformed into 1H2N and 2H1N. These compounds are then decarboxylated to 1,2DHN, which undergoes ring cleavage to form 2CCA by IDDs. Furthermore, 2CCA is converted to PA and SA, with PA being partially transformed into SA by the fungus (Fig. 8; Fig. S2). A similar metabolic pathway has been reported in microalgae, where PA is decarboxylated to SA (33). Finally, SA is decarboxylated to CAT, which is subsequently ring-cleaved to *cis*,*cis*-muconate, entering the β-ketoadipate pathway (34). In conclusion, white-rot fungi possess diverse metabolic routes for PHEN degradation.

The current study is the first to demonstrate the oxidative decarboxylation of 1H2N and 2H1N to 1,2DHN catalyzed by FPMO11 from a eukaryotic fungus. 1H2N is formed as an intermediate during the bacterial degradation of PHEN. The partially purified 1H2N hydroxylase from *Alcaligenes* sp. PPH was highly specific for 1H2N and failed to show activity with other hydroxynaphthoate analogs, including SA (35). The function and crystal structure of NahG from the PAH-degrading bacterium *P. putida* G7 have been previously elucidated (23). We generated a recombinant FPMO11 and investigated its catalytic efficiency (*k*_cat_/*K*_m_). Our results indicate that the catalytic efficiency (*k*_cat_/*K*_m_) of NahG (7.1 min**^−^**^1^mM**^−^**^1^; [23]) toward SA was slightly higher than that of FPMO11 (6.4 min**^−^**^1^mM**^−^**^1^). In contrast, the *k*_cat_/*K*_m_ of FPMO11 for 1H2N was significantly higher than that of NahG (Table 1; Fig. S8). It has been shown earlier that the putative general base, His226, that promotes substrate activation in NahG is structurally equivalent to His211 and His213 in the 6-hydroxynicotinate 3-monooxygenase NicC (36) and 3-hydroxybenzoate 6-hydroxylase 3HB6H (37), respectively. In the putative three-dimensional structure of FPMO11 generated using AlphaFold2 (Fig. 5A) in our study, we also found that the general base, His238, was conserved (Fig. 5C). Thus, the binding site for SA derivative was accurately predicted by the AlphaFill software (Fig. 6). The 1-hydroxyl group of 1H2N formed a hydrogen bond with His238, suggesting that this residue was the general base catalyst at the active site (Fig. 5C). The cavity at the substrate-binding site of FPMO11 is larger than that of NahG (Fig. 5 and 6). This also explains the differences in the catalytic properties of these FPMOs. Compared to NahG from *P. putida* G7, a large active-site pocket in FPMO11 is accessible to SA derivatives like 1H2N, 2H1N, 2H3PB, and 2H6PB composed of two aromatic rings (Fig. 5 and 6). In contrast, the narrow active-site pocket in NahG may be advantageous for its high reactivity with SA. These observations support the notion that the cavity of the active site pocket of FPMOs plays an important role in determining the substrate specificity for substrate decarboxylation. Further research using structural analysis is warranted to confirm this hypothesis.

The cleavage of the aromatic ring is one of the most important reactions in PHEN degradation. IDDs cleave the aromatic ring between two hydroxyl groups, whereas extradiol dioxygenases (EDDs) cleave the aromatic ring between one hydroxylated carbon and another non-hydroxylated carbon (38). Previously, EDD-type 1,2DHN dioxygenase, such as NahC, was identified in *P. putida* (38), and the enzyme catalyzes the *meta*-cleavage of 1,2DHN to 2-hydroxybenzalpyruvic acid (39). In some bacteria, 1,2DHN is *ortho*-cleaved to 2CCA (20, 22). However, the enzymes involved in the *ortho*-cleavage of 1,2DHN remain obscure in bacteria. IDD1 and IDD2 catalyze the *ortho*-cleavage of 1,2DHN (Fig. 7). This is the first study to identify and characterize 1,2DHN dioxygenase activities in members of the IDD superfamily. These findings underscore the unique and broad substrate spectrum of IDDs, making them attractive candidates for biotechnological applications.

In addition to 1,2DHN, dihydroxylated PHENs, such as 3,4 Dl and/or 1,2PDL, may be cleaved by IDDs. Because 3,4PDL and 1,2PDL are not commercially available, it is unclear whether dihydroxylated PHENs are cleaved by IDDs. Further studies using chemically synthesized 3,4PDL and 1,2PDL are required to confirm this pathway. If 3,4PDL and 1,2PDL are cleaved by IDDs, then these enzymes are likely to play a more important role in PHEN degradation by white-rot fungi. Additionally, SA is decarboxylated to CAT by FPMO11 (Fig. 3), after which CAT undergoes ring cleavage mediated by IDDs (33). These findings suggest that FPMO11 and IDD participate in multiple steps of the PHEN degradation pathway in the fungus (Fig. 8).

In conclusion, the present study demonstrates that FPMO11, a flavoprotein monooxygenase from *P. chrysosporium*, catalyzes the oxidative decarboxylation of the hydroxynaphthoates 1H2N and 2H1N. To the best of our knowledge, this is the first study to identify and characterize the enzyme with 1H2N and 2H1N monooxygenase activities among the members of the FPMO superfamily. We also identified two intradiol dioxygenases in *P. chrysosporium*, IDD1 and IDD2, that catalyze the ring cleavage reaction of 1,2DHN, which is the decarboxylation product of hydroxynaphthoates. The study reveals that FPMO11 and IDDs, the enzymes used by white-rot fungus *P. chrysosporium* to degrade PHEN, have unique substrate-specificity spectra, making them promising biotechnological candidates.

## MATERIALS AND METHODS

### Chemicals and reagents

PHEN and 1,2DHN were purchased from Wako Pure Chemical Industries (Osaka, Japan). 1H2N, 2H1N, 1,2DHN, SA, 2,3DHB, 2,4DHB, GA, 4CSA, 2H3PB, 2PL, 3PL, 4PL, 9PL, and DPA were purchased from Tokyo Chemical Industry Co. Ltd. (Tokyo, Japan). 2H6PB was purchased from BLD Pharmaceuticals (Shanghai, China). All the chemicals were of analytical grade. Deionized water was obtained using a Milli-Q system (Merck Millipore, Billerica, MA, USA).

### Strains, cultures, and media

*P. chrysosporium* (ATCC 34541), which has been renamed as *P. chrysosporium* (40), was maintained in high carbon low nitrogen (HCLN) medium (pH 4.5) containing the following constituents per liter of distilled H_2_O: KH_2_PO_4_, 0.2 g; MgSO_4_⋅7H_2_O, 0.05 g; CaCl_2_, 0.01 g; mineral solution, 1 mL; and vitamin solution, 0.5 mL. The mineral solution contained the following constituents per liter of distilled H_2_O: nitrilotriacetate, 1.5 g; MgSO_4_⋅7H_2_O, 3.0 g; MnSO_4_⋅H_2_O, 0.5 g; NaCl, 1.0 g; FeSO_4_⋅7H_2_O, 100 mg; CoSO_4_, 100 mg; CaCl_2_, 82 mg; ZnSO_4_, 100 mg; CuSO_4_⋅5H_2_O, 10 mg; AlK(SO_4_)_2_, 10 mg; H_3_BO_3_, 10 mg; and NaMoO_4_, 10 mg. The vitamin solution contained the following constituents per liter of distilled H_2_O: biotin, 2 mg; folic acid, 2 mg; thiamine⋅HCl, 5 mg; riboflavin, 5 mg; pyridoxine⋅HCl, 10 mg; cyanocobalamine, 0.1 mg; nicotinic acid, 5 mg; dL-calcium pantothenate, 5 mg; *p*-aminobenzoic acid, 5 mg; thioctic acid 5 mg (41, 42). The medium was also supplemented with 28 mM d-glucose and 1.2 mM (HCLN) ammonium tartrate as the carbon and nitrogen sources, respectively, and was buffered with 20 mM sodium 2,2-dimethylsuccinate (pH 4.5), as described previously (40, 41). For the experiments, fungal conidia were inoculated in 30 mL of HCLN medium (pH 4.5) in a 300 mL Erlenmeyer flask and incubated at 37°C in stationary culture.

### PHEN metabolism by fungal cells

After a 2-day preincubation in 30 mL of HCLN medium, PHEN was added at a final concentration of 0.5 mM. After additional incubation for 2-, 4-, 8-, 10-, and 14-days, PHEN and its conversion products in the culture were analyzed using an LC-20AD (Shimadzu Corporation, Kyoto, Japan) equipped with an AQ-C18 column (250 mm × 4.6 mm i.d. × 5 µm pore size; GL Sciences Inc., Tokyo, Japan) with a linear gradient of distilled water and acetonitrile (100:0 to 20:80) for 5 min, isocratic elution (20:80) for 5 min, at a flow rate of 1.0 mL/min. PHEN and its conversion products were identified using GC-MS following extraction with ethyl acetate (three times), evaporation, and TMS derivation using *N*,*O*-bis(TMS)trifluoroacetamide/pyridine (2:1, [vol/vol]).

### Construction of the gene expression system

Full-length *P. chrysosporium fpmo11* gene was PCR amplified using the primer combinations specified in Fig. S2, using a DNA Thermal Cycler 2400 (Takara Bio, Otsu, Japan), with the following PCR cycle: 30 cycles of denaturing at 98°C for 10 s, annealing at 65°C for 5 s, and extension at 68°C for 5 s. PCR products were separated on 1% agarose gels, stained with ethidium bromide, and visualized using Molecular Imager FX (Bio-Rad, Hercules, CA, USA). The amplified *fpmo11* gene was inserted into the pET21a vector (Invitrogen, Carlsbad, CA, USA). The full-length *nahG* gene was synthetically produced by Eurofins Genomics and subsequently inserted into the pET21a vector. The recombinant plasmids were transformed into *E. coli* BL21 (Invitrogen) using the heat-shock method, and transformants were selected based on ampicillin resistance in lysogeny broth (LB). The identity of the genes in the recombinant plasmids was verified by DNA sequencing.

### Heterologous expression and purification of FPMOs

*E. coli* BL21 cells harboring each of the *fpmo11* and *nahG* gene expression plasmids were grown at 37°C with constant shaking in LB supplemented with 100 µg/mL ampicillin until the optical density of the cultures at 600 nm reached 0.6. FPMO expression was induced by adding 100 µM isopropyl β-D-1-thiogalactopyranoside and 100 µM riboflavin to the medium, and the cultures were incubated further up to 24 h at 28°C. Cells were harvested by centrifugation (3,000 × *g* at 20°C for 5 min), and the cell pellet was resuspended in buffer A (20 mM 2-[4-(2-hydroxyethyl)−1-piperazinyl]ethanesulfonic acid (HEPES, pH 7.4), 10% [wt/vol] glycerol, and 1 mM phenylmethylsulfonyl fluoride). Cells were lysed by sonication (5 × 30 s) using a Q700 Sonicator (Qsonica, Melville, NY, USA). After centrifugation (15,000 × *g* at 4°C for 15 min) to remove the insoluble debris, the supernatant was collected.

For protein purification, the crude lysate was loaded onto a nickel affinity column (Cytiva, Marlborough, MA, USA) equilibrated with buffer A at 4°C. The column was washed with buffer A, and the proteins were eluted with a 0–0.3 M imidazole gradient in buffer A. Yellow fractions containing the overexpressed FPMO were collected and directly loaded onto a Superdex 200 HR 10/30 column (Cytiva) equilibrated with buffer A. The resulting eluate contained the purified recombinant proteins. Protein size was determined by SDS-PAGE. The absorption spectra were obtained using SpectraMax (Molecular Devices, San Jose, CA, USA).

### FPMO enzyme assays

FPMO activity was determined as previously described (43–45), with slight modifications. Briefly, the activity and substrate specificity of FPMO11 and NahG were determined in reaction mixtures (0.5 mL) containing 0.2 µM enzyme, 200 µM NADPH, and 5 µL of substrate solution (0–600 mM in dimethyl sulfoxide) in 50 mM HEPES buffer (4-[2-hydroxyethyl]−1-piperazineethanesulfonic acid, pH 7.0 or 8.0, respectively). The reaction was initiated by the addition of the substrate, and the decrease in absorbance at 340 nm was monitored using a spectrophotometer. The reaction mix was incubated at 30°C for 60 min, and then, 20 µL of 1 M HCl was added to stop the reaction. The background rate of NADPH consumption in the reaction mixture without the substrate was subtracted from the initial rates observed with various substrates. The residual substrate and reaction products were analyzed using LC-20AD with a linear gradient of distilled water and acetonitrile for 15 min at a flow rate of 1.0 mL/min. The reaction products were identified using GC-MS after extraction with ethyl acetate at pH 2, drying over MgSO_4_, evaporation under N_2_, and TMS derivatization, as described above. The optimum temperature for the FPMO11 reaction was determined by measuring the activity over a range of 10°C–70°C. The optimal pH was determined using 50 mM sodium acetate (pH 3.0–6.0), 50 mM HEPES (pH 6.0–8.5), and 50 mM Tris-HCl (pH 8.5–9.0).

### IDD enzyme assays

Previously, we prepared and purified the IDDs, IDD1, and IDD2 (30). IDD activity was determined as previously described (30), under air-saturated conditions by measuring the O_2_ consumption during the enzyme reaction polarographically using Oxytherm + R (Hansatech Instruments, Norfolk, UK). Briefly, the activity of IDDs toward 1,2DHN was determined in reaction mixtures (0.5 mL) containing 2 µM IDDs and 5 µL substrate solution (0–600 mM in acetonitrile) in 50 mM Tris-HCl buffer (pH 8.0). The reaction was initiated by adding the substrate, and O_2_ consumption in the reaction mixture was monitored using a Clark O_2_ electrode. Superoxide dismutase (6 µg) was added to the reaction mixture to avoid rapid autoxidation of the substrates. The background rate of O_2_ consumption in the reaction mixture without the substrate was subtracted from the observed rates for the various substrates. The apparent kinetic parameters *K*_m_ and *k*_cat_ were calculated by fitting the obtained initial rates using the Michaelis-Menten equation in the Origin version 6.0 software (OriginLab, Northampton, MA, USA). After an enzymatic reaction for 60 min at 37°C, the residual substrate and reaction products were analyzed and identified by GC-MS after extraction with ethyl acetate at pH 2, drying over MgSO_4_, evaporation under N_2_, and TMS derivatization, as described above.

### Structural analysis of FPMO11

The FPMO11 structural model was predicted using AlphaFold 2 (28). The crystal structure of NahG liganded with SA was obtained from the Protein Databank (PDB ID: 5EVY). Based on the obtained FPMO11 structural data, the binding patterns of ligands and cofactors were predicted using AlphaFill (https://alphafill.eu) (29). Ligand docking models were analyzed using PyMOL software.

### Analytical methods

GC-MS was performed at 70 eV using a GCMS-QP2010 (Shimadzu, Kyoto, Japan) apparatus equipped with a 30 m fused silica column (DB-5, J & W Scientific, Folsom, CA, USA). The oven temperature was programed to ramp from 80°C to 320°C at 8 °C /min, with an injection temperature of 280°C. The substrate conversion products were identified by comparing their respective GC retention times and mass fragmentation patterns with those of authentic standards (46, 47). The compounds for which authentic standards were unavailable were identified by comparing their mass data with those available from the National Institute of Standard Technology Mass Spectral Library.

## Data Availability

All data generated or analyzed during this study are included in this published article and its supplemental files.

## SUPPLEMENTAL MATERIAL

Supplemental material aem.01574-24-s0001.docx Figures S1 to S9; Tables S1 and S2.

ASM does not own the copyrights to Supplemental Material that may be linked to, or accessed through, an article. The authors have granted ASM a non-exclusive, world-wide license to publish the Supplemental Material files. Please contact the corresponding author directly for reuse.
