# Supplementary material for: Phenanthrene degradation by a flavoprotein monooxygenase from Phanerodontia chrysosporium
Source: Appl Environ Microbiol. 2025 Feb 3;91(3):e01574-24. doi: 10.1128/aem.01574-24 (PMC11921375; doi:10.1128/aem.01574-24)
Supplement: Supplemental material — Figures S1 to S9; Tables S1 and S2. [file aem.01574-24-s0001.docx]

**Supplemental materials**

**Phenanthrene degradation by a flavoprotein monooxygenase from *Phanerodontia chrysosporium***

**Running title: Phenanthrene degradation by *P. chrysosporium***

Mika Hayasaka, Link Hamajima, Yuki Yoshida, Reini Mori, Hiroyuki Kato, Hiromitsu Suzuki, Ryoga Tsurigami, Takaaki Kojima, Masashi Kato, and Motoyuki Shimizu#

Faculty of Agriculture, Meijo University, Nagoya, Japan

#Address correspondence to Motoyuki Shimizu (ORCID; 0000-0002-6907-6367), [moshimi@meijo-u.ac.jp](mailto:moshimi@meijo-u.ac.jp)

**FIG. S1 Total ion chromatograms and mass spectra of the metabolites from PHEN (A-K), 1H2N, and 2H1N (L-S).** The trimethylsilyl (TMS)-derivatized metabolites were analyzed using gas chromatography mass spectrometry (GC-MS). Total ion chromatograms of the metabolites from phenanthrene (PHEN) (A, G, I) or 1H2N (L, N, P, R). The mass spectra (B, 4-phenanthrol (4PL); C, 9-phenanthrol (9PL); D, 3-phenanthrol (3PL); E, 2-phenanthrol (2PL); F, diphenic acid (DPA); H, 3,4-phenanthrenediol (3,4PDL); J, 1-hydroxy-2naphthoate (1H2N); K, 2-hydroxy-1naphthoate (2H1N); M, 1,2-dihydrocynaphtharene (1,2DHN); O, 2-carboxycinnamic acid (2CCA); Q, phthalic acid (PA); S, salicylic acid (SA)) of the metabolites were obtained from the GC peaks appearing at retention times 45.3 min (B), 46.5 min (C), 47.3 min (D), 48.3 min (E), 46.0 min (F), 50.0 min (H), 43.2 min (J), 43.6 min (K), 37.3 min (M), 42.9 min (O), 34.4 min (Q) and 28.0 min (S). The experiment was performed three times, and representative results are shown.

**FIG. S2 Fungal metabolism of 2-CCA, PA, and SA.**

(A, C, E) Time course of 2-carboxycinnamic acid (2CCA), phthalic acid (PA), and salicylic acid (SA) conversion. After a 2-day pre-incubation, 2CCA (A), PA (C), and SA (E) were added to a final concentration of 0.5 mM. (B, D, F) The metabolites of 2CCA (B), PA (D), or SA (F) in the culture were identified using gas chromatography mass spectrometry (GC-MS) with authentic standards as references. After 14 days of incubation with 2CCA, PA, or SA, metabolites of 2CCA, PA, or SA were identified using GC-MS. 2-carboxycinnamic acid (2CCA, ●); phthalic acid (PA, ▲); salicylic acid (SA, ⯁); catechol (CAT, ■). Data are presented as mean values of three independent experiments. The standard errors were <16%.

**FIG. S3 Proposed metabolic pathway of phenanthrene by the white-rot fungus *Phanerodontia chrysosporium*.**

**Fig. S4 Amino acid sequence alignment of FPMO11 homologs.**

The sequences of flavoprotein monooxygenase 11 (FPMO11; Protein ID 6385018), salicylate 1-monooxygenase (NahG) from *Pseudomonas putida* (UniProt identifier P23262), ShyA from *Aspergillus niger* (A2GWH1), and SalA from *A. nidulans* (Q9HFQ8) are shown. The protein IDs for *P. putida*, *A. niger*, and *A. nidulans* were obtained from UniProt Knowledgebase (http://beta.uniprot.org), and that for *P. chrysosporium* was obtained from JGI Genome Portal (https://mycocosm.jgi.doe.gov/Phchr4_2/Phchr4_2.home.html). Conserved amino acid residues are highlighted in black. The residue (H238 of FPMO11), highlighted in red, is responsible for substrate deprotonation and catalytic activity. Phenylalanine residues (F242 and F255) that increase the hydrophobicity of the substrate pocket, and charged residues (H338 and D397) involved in the interaction with the substrate are shown in red boxes. The amino acid residues involved in FAD retention are indicated in yellow boxes. Sequences were aligned using ClustalW (https://www.genome.jp/tools-bin/clustalw).

**
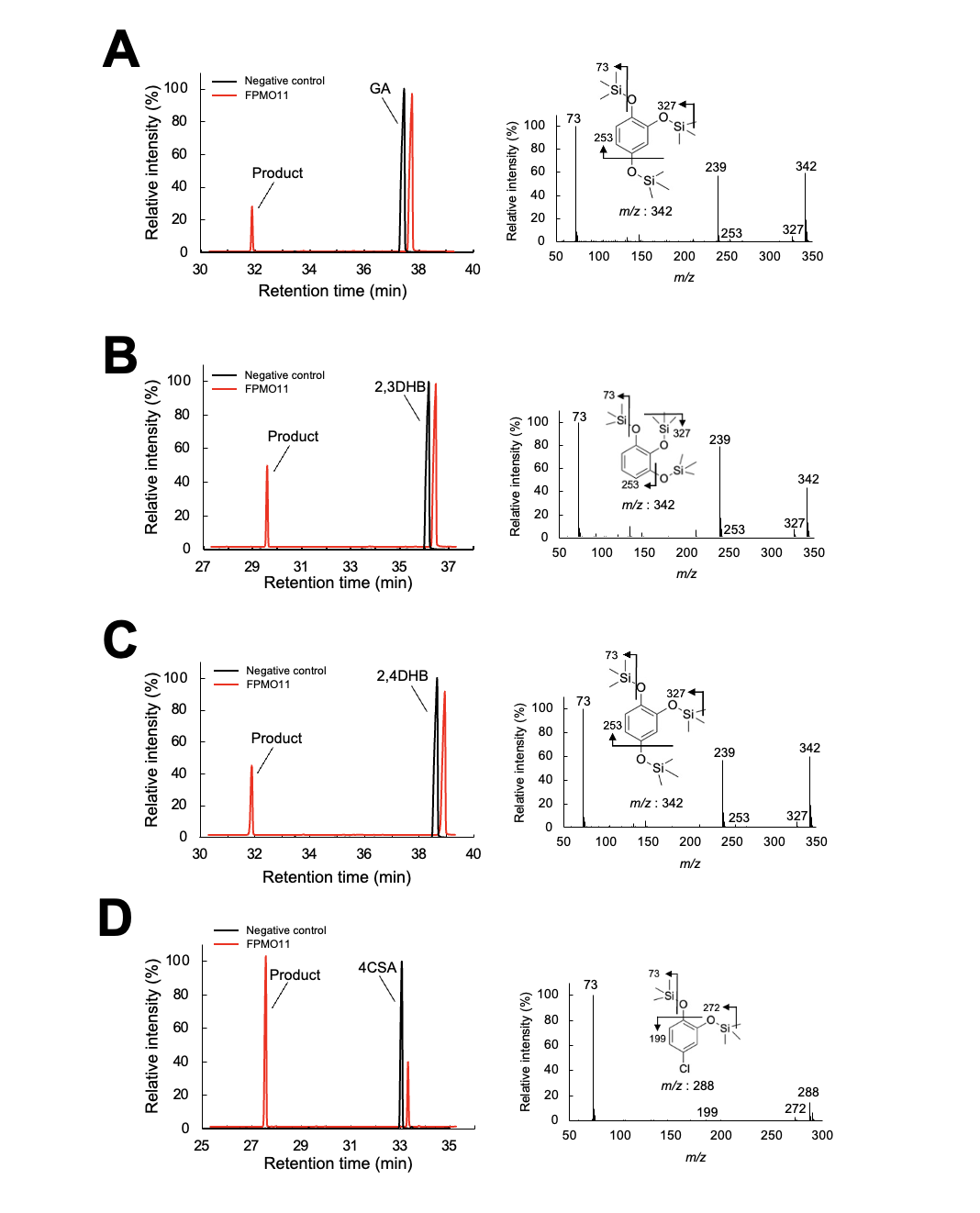
**

**
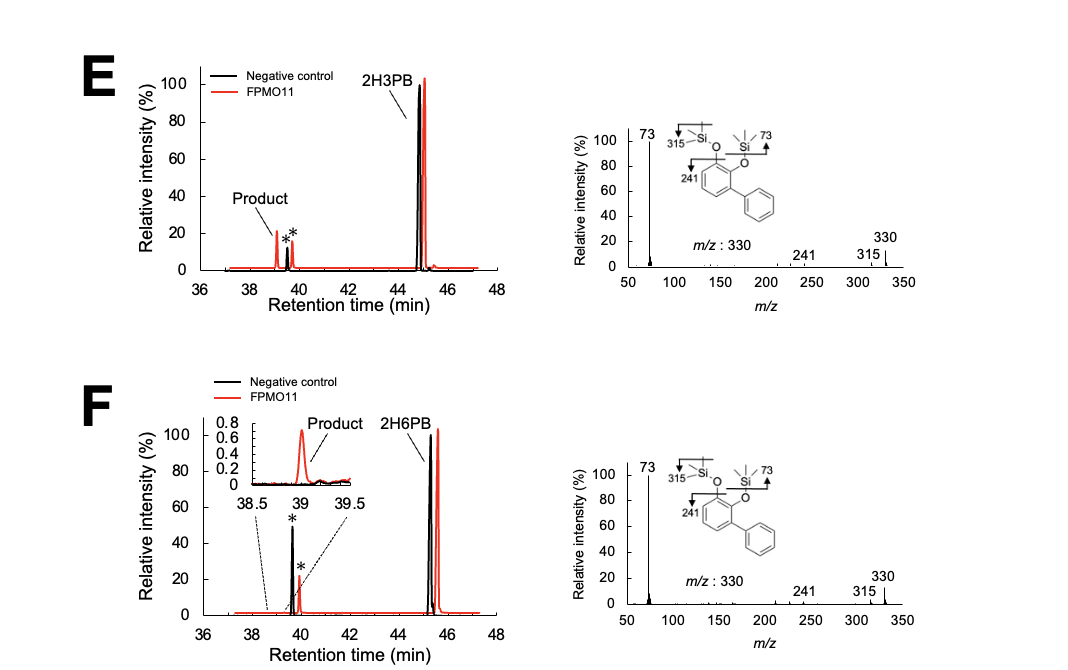
FIG. S5 Total ion chromatograms and mass spectra of reaction products generated by flavoprotein monooxygenase (FPMO)11 from GA (A), 2,3DHB (B), 2,4DHB (C), 4CSA (D), 2H3PB (E), and 2H6PB (F) as substrates.**

The trimethylsilyl (TMS) derivatization products from the reaction with the substrates were analyzed using gas chromatography mass spectrometry (GC-MS). The mass spectra (A, 1,2,4-trihydroxybenzene (1,2,4THB); B, pyrogallol (PG); C, 1,2,4THB; D, 4-chlorocatechol (4CCAT); E, biphenyl-2,3-diol (B2,3D); F, biphenyl-2,3-diol (B2,3D); from reaction products were obtained from the GC peaks appearing at retention times of 32.1 min (A, C), 29.6 min (B), 27.5 min (D), and 39.0 min (E, F), respectively. The asterisks indicate contaminants. The results are representative, and the experiments were performed three times.

**
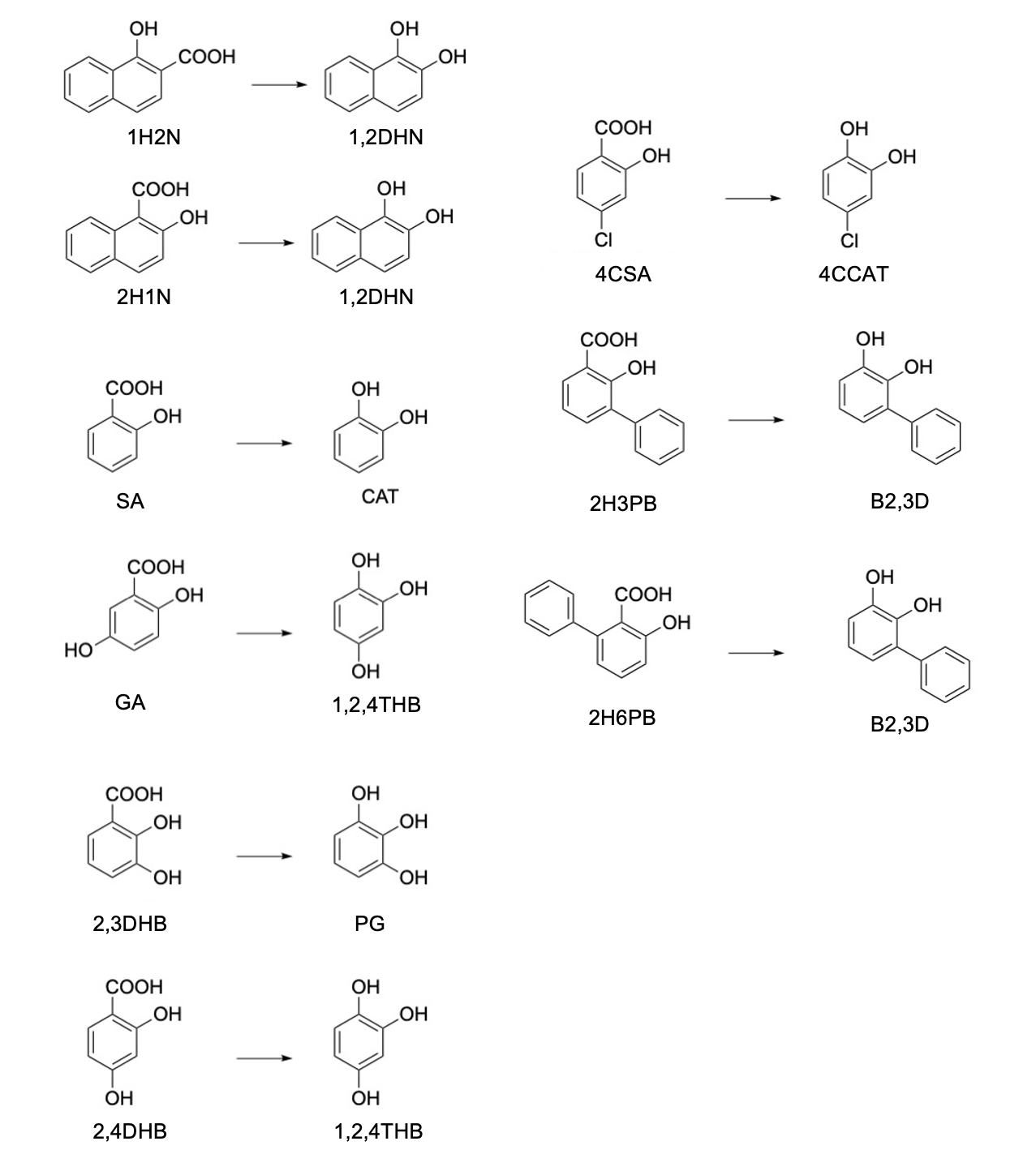
FIG. S6 Decarboxylation of nine SA derivatives by flavoprotein monooxygenase 11.**

**FIG. S7 SDS-PAGE analysis of recombinant NahG.**

SDS-PAGE analysis of purified NahG. Lane 1: NahG; lane M: protein molecular mass marker.


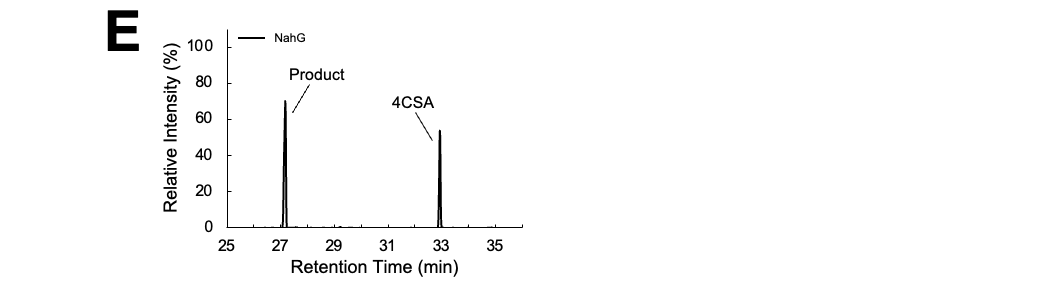

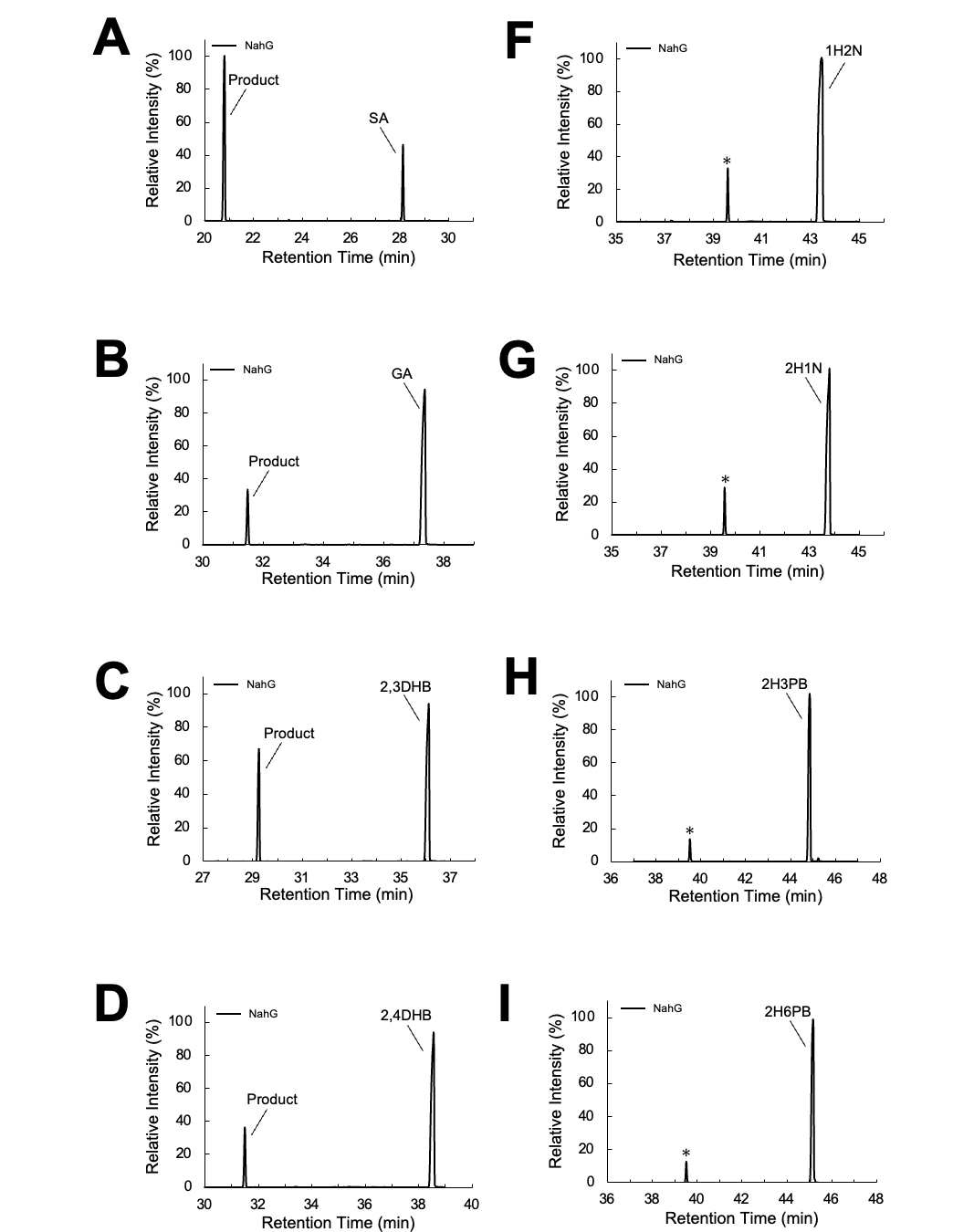


**FIG. S8 Total ion chromatograms of substrates and reaction products generated by salicylate 1-monooxygenase (NahG) from SA (A), GA (B), 2,3DHB (C), 2,4DHB (D), 4CSA (E), 1H2N (F), 2H1N (G), 2H3PB (H) and 2H6PB (I) as substrates.**

The trimethylsilyl (TMS) derivatization products from the reaction with the substrates were analyzed using gas chromatography mass spectrometry (GC-MS). The asterisks indicate contaminants. The results are representative, and the experiments were performed three times.

**FIG. S9 SDS-PAGE analysis of recombinant IDD1 and IDD2.**

SDS-PAGE analysis of purified intradiol dioxygenase (IDD)1 and IDD2. Lane 1, IDD1; lane 2, IDD2; lane M, protein molecular mass markers.

**Table S1 Kinetic parameters of IDD1 and IDD2 towards 1,2DHN**

|  | **IDD1** | | | **IDD2** | | |
| --- | --- | --- | --- | --- | --- | --- |
| **Substrate** | ***K_m_* (mM)** | ***k_cat_* (s^-1^)** | ***k_cat_/K_m_***  **(s^-1^/mM)** | ***K_m_* (mM)** | ***k_cat_* (s^-1^)** | ***k_cat_/K_m_***  **(s^-1^/mM)** |
| 1,2DHN | 0.48 ± 0.018 | 4.6 ± 1.1 | 9.5 | 0.52 ± 0.18 | 0.56 ± 0.21 | 1.1 |

The activity levels of intradiol dioxygenase (IDD)s were determined in the reaction mixtures (0.5 mL) containing 2 µM IDDs and 0.1–5 µL of substrate solutions (0–600 mM in dimethylsulfoxide) in 50 mM Tris-HCl buffer (pH 8.0) at 40 °C. The initial velocity after 10 s of addition of each substrate was used to calculate the apparent kinetic parameters. Data are presented as mean ± standard error of three experiments**.**

**Table S2 Oligonucleotide primers for cloning for recombinant protein expression**

| FPMO11-f | 6385018 | 5’-GTGGACAGCAAATGGGTCGCGGATCCCCGGAGCGCGCGATGCGAGT-3’ |
| --- | --- | --- |
| FPMO11-r |  | 5’-TGTCGACGGAGCTCGAATTCGGATCCGCGCTTGAAGCTTTGAAGA-3’ |

Gene-specific primers were designed based on genomic sequence data (https://mycocosm.jgi.doe.gov/Phchr4_2/Phchr4_2.home.html).
